# Effect of Pediatric Influenza Vaccination on Antibiotic Resistance, England and Wales

**DOI:** 10.3201/eid2601.191110

**Published:** 2020-01

**Authors:** Chungman Chae, Nicholas G. Davies, Mark Jit, Katherine E. Atkins

**Affiliations:** London School of Hygiene and Tropical Medicine, London, UK (C. Chae, N.G. Davies, M. Jit, K.E. Atkins);; Public Health England, London (M. Jit); University of Edinburgh, Scotland, UK (K.E. Atkins)

**Keywords:** antibacterial agents, bacterial infections, child, cost savings, drug resistance, antimicrobial resistance, microbial, England, influenza vaccines, influenza, human, mathematical model, prescriptions, primary healthcare, referral and consultation, United Kingdom, vaccination, vaccines, LAIV, attenuated, Wales, respiratory infections, viruses, vaccine-preventable diseases

## Abstract

Vaccines against viral infections have been proposed to reduce prescribing of antibiotics and thereby help control resistant bacterial infections. However, by combining published data sources, we predict that pediatric live attenuated influenza vaccination in England and Wales will not substantially reduce antibiotic consumption or adverse health outcomes associated with antibiotic resistance.

Antibiotic use drives the spread of antibiotic resistance. A considerable proportion of antibiotic prescriptions are prescribed unnecessarily for conditions that are either self-limiting or nonbacterial in etiology (*1*). Because influenza is often treated inappropriately with antibiotics, expanding access to influenza vaccines has been proposed as a means of reducing unnecessary prescribing and preventing resistant infections (*2*).

In 2013, England and Wales began rolling out the live attenuated influenza vaccine (LAIV) for 2–16-year-old children (*3*). Here, we estimate the potential effect on antibiotic prescribing and antibiotic resistance.

## The Study

We assumed that some influenza cases lead to general practitioner (GP) consultations and some GP consultations lead to antibiotic prescriptions. Our age-stratified analysis focused on community antibiotic use as the driver of resistance, because hospitalizations for influenza are rare relative to GP consultations (*4*).

To estimate the influenza-attributable consultation rate, we used a previous time-series statistical attribution covering the 1995–2009 influenza seasons in the United Kingdom (*5*), yielding a population-wide average of 14.7 influenza-attributable GP consultations per 1,000 person-years (Table 1). For our uncertainty analysis (Appendix Table), we used a lower estimate of 11.8 per 1,000, from a longitudinal study of the 2006–2011 influenza seasons in England (*6*), and a higher estimate of 21.4 per 1,000, from a time-series statistical analysis of the 2000–2008 influenza seasons in England and Wales (*4*).

**Table 1:** Projected effect of pediatric LAIV on antibiotic prescription rates, England and Wales*

Age group	Influenza-attributed consultation rate†	Prescriptions per consultation	Direct prescribing rate reduction, unmatched‡	Direct prescribing rate reduction, matched‡	Overall LAIV effectiveness§	Overall prescribing rate reduction¶
0–6 mo	29.7 (23.7–35.9)	0.597 (0.474–0.719)	–	–	0.574 (0.501–0.651)	10.2 (7.03–13.5)
6 m–4 y	29.7 (23.7–35.9)	0.597 (0.474–0.719)	7.46 (5.31–9.64)	12.4 (8.85–16.1)	0.663 (0.618–0.714)	11.8 (8.31–15.4)
5–14 y	22.1 (17.6–26.7)	0.588 (0.466–0.708)	5.46 (3.89–7.06)	9.11 (6.48–11.8)	0.754 (0.709–0.794)	9.81 (6.97–12.8)
15–44 y	12.8 (10.2–15.4)	0.676 (0.536–0.814)	3.64 (2.59–4.70)	6.06 (4.31–7.83)	0.446 (0.394–0.502)	3.86 (2.66–5.09)
45–64 y	12.4 (9.84–14.9)	0.805 (0.639–0.970)	–	–	0.423 (0.374–0.484)	4.22 (2.90–5.58)
>65 y	12.2 (9.67–14.7)	0.857 (0.680–1.03)	–	–	0.477 (0.397–0.561)	4.97 (3.34–6.68)
Overall	14.7 (11.7–17.7)	0.726 (0.576–0.875)	5.80 (4.13–7.49)	9.86 (7.01–12.9)	0.494 (0.446–0.549)	5.32 (3.74–7.00)

*All estimates reported as mean (95% highest density interval). LAIV, live attenuated influenza vaccine; –, age group not subject to pediatric LAIV. †Per 1,000 person-years in England and Wales. ‡Reduction in antibiotic prescriptions among vaccinees per 1,000 vaccine recipients, not accounting for herd immunity, presented separately for unmatched and matched seasons. §Reduction in influenza cases assuming a 50% uptake among children 2–16 years of age, accounting for herd immunity. ¶Per 1,000 person-years in England and Wales, accounting for herd immunity.

We estimated that 726 antibiotic prescriptions are written for every 1,000 influenza-attributable GP consultations (*5*). For our uncertainty analysis, we used a lower estimate of 313 per 1,000, derived from electronic health records of prescriptions within 30 days of a consultation for influenza-like illness (ILI) or acute cough in England during 2013–2015 (*7*).

We assumed that LAIV prevents 49% of symptomatic influenza cases on average, using a previously published mathematical model of pediatric LAIV in England and Wales, which assumes 50% uptake and either 70% (matched-year) or 42% (unmatched-year) efficacy among 2–16-year-olds (*3*). This reduction is consistent with a pilot study comparing consultation rates in treatment with control areas before and after LAIV rollout (*8*). For our uncertainty analysis, we used lower and higher estimates of 32% and 63% fewer influenza cases from the same model, assuming an uptake of 30% and 70%, respectively.

To predict the healthcare benefits of reducing unnecessary prescribing, we used linear regression with a country’s rate of primary-care antibiotic use as the predictor variable and previously published 2015 estimates of adverse health outcomes associated with 16 resistant bacterial strains across European countries (*9*) as the response variables. We adopted a published cost estimate of $1,415 per resistant infection (2016 USD) (*10*), adjusted for inflation and healthcare purchasing power parity to £520 (2015 GBP).

We used Monte Carlo sampling to explore uncertainty across estimates for consultation rate, prescribing rate, and LAIV effectiveness, weighting age groups by using 2015 demographic data for England and Wales. (Analysis code and data at http://github.com/nicholasdavies/laiv_amr_ew; additional details in the Appendix.)

We found that pediatric LAIV has the potential to reduce antibiotic consumption by 5.3 (95% highest density interval [HDI] 3.7–7.0) prescriptions per 1,000 person-years (Table 1) across the population of England and Wales, or 0.8% of the antibiotic dispensation rate for primary care in England and Wales in 2015. For comparison with secular trends, this rate has fallen by 2.5% each year during 2012–2018 in England (Appendix Figure). Focusing on vaccine recipients only, we estimated that the direct effectiveness of LAIV on antibiotic consumption is 5.8 (95% HDI 4.1–7.5) fewer prescriptions per 1,000 person-years in unmatched years and 9.9 (95% HDI 7.0–13) in matched years.

**Figure 1:**
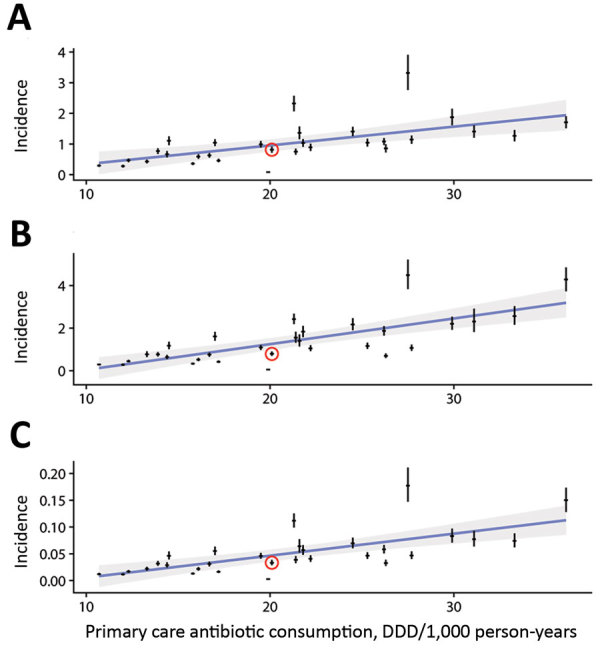
Estimated incidence of adverse health outcomes resulting from antibiotic-resistant infections, plotted against the overall antibiotic consumption in primary care settings in 30 countries in Europe, 2015. A) Antibiotic-resistant cases/1,000 person-years; B) attributable DALYs/1,000 person-years; C) attributable deaths/1,000 person-years. Red circles indicate datapoints for the United Kingdom; error bars indicate 95% CIs. Blue lines indicate linear regressions; gray shading indicates 95% confidence regions for linear regressions. DALYs, disability-adjusted life years; DDD, defined daily dose.

**Figure 2:**
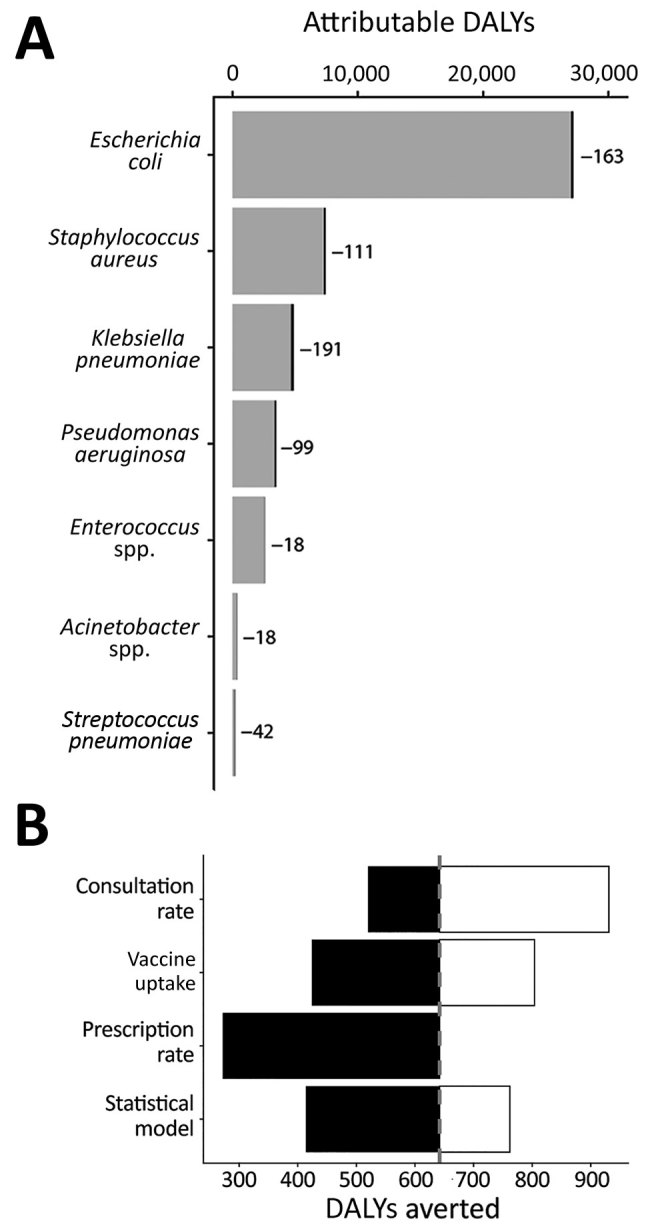
Effect of pediatric LAIV on adverse health outcomes attributable to antibiotic-resistant bacterial strains, England and Wales. A) Estimated DALYs attributable to resistant infections averted by pediatric LAIV, stratified by causative pathogen. The entire width of each bar is the current number of DALYs; potential reductions are highlighted in black and reported next to each bar. B) One-way uncertainty analysis, showing the effect on DALYs averted, of alternative assumptions concerning the rate of influenza-attributable general practitioner consultations, the pediatric uptake of LAIV, the rate of antibiotic prescribing per general practitioner consultation, and how the effect of prescribing on adverse health outcomes associated with resistance is attributed (additional details in Methods and Appendix). DALYs, disability-adjusted life years; LAIV, live attenuated influenza vaccine.

Although 0.8% is a small decrease in antibiotic use, it might appreciably improve the cost-effectiveness of pediatric LAIV if the healthcare costs of resistance are substantial enough (Figure 1). We estimated that LAIV has the potential to reduce resistance-attributable disability-adjusted life years (DALYs) by 642, cases by 432, and deaths by 22 per year in England and Wales (Table 2); averted DALYs were spread relatively evenly across the 7 causative pathogens analyzed (Figure 2, panel A). We estimated a yearly cost saving of £224,000 for averted resistant infections. Compared with the projected incremental cost (program cost minus healthcare saving) of pediatric LAIV at £63.6 million, and its projected effect of saving 27,475 quality-adjusted life years and averting 799 deaths yearly (*3*), accounting for resistance will not substantially increase the cost-effectiveness of pediatric LAIV in this setting. Our uncertainty analysis (Figure 2, panel B) identified the consultation rate as having the greatest influence over the effect of LAIV on resistance-associated adverse health outcomes.

**Table 2:** Projected effect of pediatric LAIV on adverse health outcomes associated with antibiotic resistance, England and Wales*

Outcome	Estimate for 2015, England and Wales	Projected reduction in outcome resulting from LAIV, mean (95% HDI)
DALYs	46,039	642 (450–842)
Cases	47,080	432 (303–566)
Deaths	1,930	22 (16–29)

*DALYs, disability-adjusted life years; HDI, highest density interval; LAIV, live attenuated influenza vaccine.

## Conclusions

Our estimates for the foreseeable reduction in antibiotic prescribing from the LAIV program in England and Wales might seem surprisingly low, given that sore throat, cough, and sinusitis together account for 53% of all inappropriate prescribing, which in turn accounts for at least 9%–23% of all prescribing in England (*1*). However, many viral and bacterial pathogens cause these symptoms. By one estimate, influenza causes only 11% of GP consultations for acute respiratory illness in England (*4*), so it might be optimistic to expect influenza vaccination to substantially reduce antibiotic use in this setting.

Our base-case estimate of 726 antibiotic prescriptions per 1,000 influenza-attributable consultations is more than double what electronic health records suggest (7). One explanation is that our estimate, derived from statistical attribution of antibiotic prescriptions to influenza circulation during 1995–2009 (*5*), feasibly includes prescribing for secondary infections such as otitis media, sinusitis, and pneumonia. Moreover, electronic health records might not reliably reflect antibiotic prescribing rates for influenza: in 1 study, only 8% of consultations for ILI resulted in influenza or ILI being medically recorded (*6*). Conversely, antibiotic use in England has declined since 1995 (by 22% during 1998–2016) (*11*). Accordingly, our base-case results should be interpreted as the maximum potential reduction by LAIV of antibiotic use.

In randomized trials, the direct effect of influenza vaccines on vaccinated children has ranged from a 44% reduction (Italy) to a 6% increase (United States) in antibiotic prescriptions over the 4-month period following vaccination, whereas estimates of the effect over entire populations (all ages, vaccinated and unvaccinated) range from 11.3 fewer prescriptions per 1,000 person-years in Ontario, Canada, to 3.9 fewer in South Africa and Senegal (Appendix). This variability might arise from differences in vaccine efficacy and coverage, population risk factors, influenza circulation, or existing patterns of antibiotic use, which make generalizing estimates across settings challenging.

The adverse health outcome estimates that we adopt (*9*) assume that resistant infections add to, rather than replace, nonresistant infections. Relaxing this assumption would further reduce the projected effect of LAIV, because some prevented resistant infections would be replaced by nonresistant infections (*12*).

Our framework estimates the effect of influenza vaccination on antibiotic resistance by using the relationship between influenza circulation and antibiotic use in England and Wales, and can be adapted to other settings for which this relationship can be quantified. An alternative approach would be to correlate LAIV uptake, rather than influenza circulation, directly with antibiotic use. Challenges with that approach include appropriately controlling for confounding factors in the relationship between vaccine uptake and antibiotic use and quantifying herd immunity. However, consistent with our approach, UK-specific empirical estimates have suggested little or no effect of LAIV uptake on prescribing: a self-controlled case-series study found that 2–4-year-old LAIV recipients took 13.5% fewer amoxicillin courses in the 6 months after vaccination (*13*), whereas an LAIV pilot study detected no difference in prescribing rates for respiratory tract infections between treatment groups (*14*). No single vaccine is likely to substantially reduce inappropriate antibiotic use in the United Kingdom.

Appendix Additional information regarding effect of pediatric influenza vaccination on antibiotic resistance, England and Wales.
