# Supplementary material for: Effect of Pediatric Influenza Vaccination on Antibiotic Resistance, England and Wales
Source: Emerg Infect Dis. 2020 Jan;26(1):138–42. doi: 10.3201/eid2601.191110 (PMC6924886; doi:10.3201/eid2601.191110)
Supplement: Appendix — Additional information regarding effect of pediatric influenza vaccination on antibiotic resistance, England and Wales. [file 19-1110-Techapp-s1.pdf]

Article DOI: https://doi.org/10.3201/eid2601.191110

Effect of Pediatric Influenza Vaccination on Antibiotic Resistance, England and Wales

Appendix

Influenza-attributable GP consultations

Base-case estimate

This estimate is derived from Table 2 of Fleming et al. (*1*), giving consultations for respiratory disease broadly defined attributable to either influenza A or B. Age groups reported by Fleming et al. differ from those used in this study, so we adapt estimates of Fleming et al. by assuming that reported rates are constant within an age group, and that half of children under 12 months old are under 6 months old.

Fleming et al. do not directly report confidence intervals in measured rates (instead, variation between flu seasons is reported), so we assume that uncertainty in the influenza-attributable GP consultation rates follows a normal distribution. We assume that the standard deviation of any consultation rate derived from this source is always S times the mean rate, where S is estimated from Figure 2 of Fleming et al. (*1*) by assuming that the width of the 95% confidence intervals on this figure are equivalent to 1.96 times the standard deviation of an associated normal distribution, and that the standard deviation of influenza-attributable GP visits is $(a^2 + b^2)^{1/2}$ where a is the standard deviation of influenza A-attributable consultations and b is the standard-deviation of influenza B-attributable consultations. S is then the mean relative standard deviation, calculated in this manner, across all study years.

Low estimate

Rates of PCR confirmed influenza are estimated from Tables S2 and S3 of Hayward et al. (*2*), taking the mean over the five winter flu seasons reported (i.e., excluding the Summer 2009 pandemic flu period). We assume that the reported rates of PCR confirmed influenza in the form B (A – C) represent a triangular distribution with B as the peak (mode) and A – C as the 95% highest density interval (using a triangular distribution rather than a normal distribution allows us

to account for skew). Then, the probability of a GP visit given PCR-confirmed illness is taken from Table S6 of the same source. We correct for low numbers by assuming a “base proportion” of 12/82 as the measured proportion of PCR confirmable influenza episodes resulting in a GP visit, which comes from the overall number of reported GP visits for 5–64-year-olds with PCR-confirmed influenza. To account for uncertainty in measurement, we draw the “base rate” of GP consultation given PCR-confirmable influenza for 5–64-year-olds from a beta distribution with parameters $\alpha = 12 + 1$, $\beta = 82 - 12 + 1$ (i.e., assuming a uniform prior); to account for the observation that this rate is higher in young children and the elderly (Table S6 of Hayward et al.), we add 0.12 to this rate for under-5s and over-65s. The annual influenza-attributable rate of GP consultation for a given age group is then the product of the PCR-confirmable influenza incidence and the rate of GP consultation given PCR-confirmable influenza.

High estimate

These are taken from Table 4 of Cromer et al. (*3*), assuming that reported 95% confidence intervals represent 1.96 times the standard deviation of a normal distribution.

Rate of antibiotic prescribing given an influenza-attributable GP consultation

Base-case estimate

This estimate is derived from Table 2 of Fleming et al. (*1*), by dividing the rate of antibiotic prescribing by the rate of influenza-attributable GP consultations, assuming a normal distribution for the final rate with the same relative standard deviation derived above (see “Base-case estimate” under “Influenza-attributable GP consultations”).

Low estimate

This estimate is derived from Table 3 of Pouwels et al. (*4*), which reports that 48% of consultations for acute cough and 29% of consultations for influenza-like illness result in a systemic antibiotic prescription within 30 days. We assume that 88.1% of influenza-attributable consultations are for ILI (hence having a 29% prescription rate) and the rest are for acute respiratory infection without fever (*2*) (hence having a 48% prescription rate), which yields an overall (crude) prescribing rate of 31.3%.

To calculate age-stratified values, we assume prescribing for under-5s is around 20% less, and for over-45s is around 20% more, than prescribing in 5–44-year-olds, consistent with

the results of Fleming et al. (*1*), Meier et al. (*5*), and Pitman et al. (*6*). That is, we draw a value d from a normal distribution with mean 0.2 and standard deviation 0.05, and assume that the relative prescribing rate for under-5s is $(1 - d)$ times the rate for 5–44-year-olds, while the relative prescribing rate for over-45s is $(1 + d)$ times the rate for 5–44-year-olds.

Impact of LAIV on rates of GP consultation

We use fitted models from Baguelin et al. (*7*) projecting the impact of LAIV on influenza cases in different age groups, assuming either a 50% vaccine uptake (base-case estimate), 30% uptake (low estimate), or 70% uptake (high estimate).

Age-stratified rates for uncertainty analysis

We summarize the base-case and uncertainty-analysis estimates of age-stratified consultation rates, prescription rates, and overall LAIV effectiveness in the Appendix Table.

Prediction of prescription rate impact on resistance-associated health burdens

Defined daily doses per prescribed antibiotic course

We assume that each prescription comprises 7 defined daily doses (DDD), as 7 days is the typical duration of antibiotic treatment for upper respiratory tract infections (*8*).

Main scenario

We use total primary care antibiotic consumption (ATC code J01C) for European countries for 2015 from the ECDC (*9*) as the predictor variable, and per-country median health burden (DALYs, cases, or deaths) attributed to each of 16 resistant strains analyzed by Cassini et al. (*10*) as the outcome variable, in a series of country-level linear regressions from which we separately predict the impact of reducing overall prescribing by a defined amount. For each country, we normalize each resistant-strain-specific health burden to the total number of bloodstream infections caused by the species in question before performing the regression to control for differences in the population and the per-capita incidence of infection between countries.

To estimate the total number of bloodstream infections caused by a given species in a given country, we begin by taking the maximum of the number of total tested isolates recorded by the ECDC for that country and species. Then we correct that figure according to the estimated population coverage for that country and species to the ECDC (i.e., an estimate of what fraction of the population is covered by the hospitals submitting resistance testing data to national surveillance programs which then report their data to the ECDC). For example, for *S. pneumoniae* infections in the United Kingdom in 2015, 1095 isolates were tested for penicillin non-susceptibility, 1077 isolates were tested for macrolide non-susceptibility, and 1060 isolates were tested for combined non-susceptibility to both penicillins and macrolides. Additionally, these isolates were reported as covering an estimated 21% of the entire population of the UK. Accordingly, we estimated the total number of bloodstream infections by *S. pneumoniae* in the UK as $\max(1095, 1077, 1060) / 0.21 = 1095 / 0.21 \approx 5214$.

An alternative method whereby health burdens were normalized to the population of each country produced similar results (a mean reduction of 714 instead of 642 DALYs, 362 instead of 432 cases, and 24 instead of 22 deaths).

Alternative scenario 1

Rather than using the overall antibiotic consumption for each country as the sole predictor in the regression model, we also built a separate series of models where we used as predictors each country's consumption of tetracyclines (J01AA), extended spectrum penicillins (J01CA), β-lactamase sensitive penicillins (J01CE), and macrolides (J01FA), as these four classes comprise the majority of antibiotics prescribed for sore throat and cough (*11*). We assume that for a given reduction in the overall prescription rate x, there is a reduction $0.0620x$ in tetracycline prescribing, $0.4752x$ in extended spectrum penicillin prescribing, $0.2793x$ in β-lactamase sensitive penicillin prescribing, and $0.1835x$ in macrolide prescribing. This predicted a smaller impact upon resistance than the main scenario (4.1) and comprises the "low-effect" statistical model for the uncertainty analysis (Figure 2B, main text).

Alternative scenario 2

We follow the same procedure as in 4.2, but if any predictor variable is negatively correlated with a resistance related health burden (i.e., the best fitting linear model suggests that decreasing use of that antibiotic would increase resistance), we remove it from the linear

regression and rerun the model, continuing this process until all predictors are positively associated with the outcome variable. If more than one variable has a negative association in a given round, all negative-association variables are removed for the next round. This predicted a larger impact upon resistance than the main scenario (4.1) and comprises the "high-effect" statistical model for the uncertainty analysis (Figure 2B, main text).

Economic calculations

To convert between U.S. and UK healthcare expenditures, we use hospital-service price level indices for health care purchasing power parity published by the OECD (*12*) (see their Figure 1).

Secular trends in antibiotic prescribing rates

Data from NHS Digital show that community antibiotic use in England has decreased by ≈2.5% per year from 2012 to 2018 (Appendix Figure).

The impact of influenza vaccination on antibiotic use in different settings

A systematic review (*13*) found that in randomized trials, the direct effect of influenza vaccines on vaccinated children has ranged from a 44% reduction in antibiotic prescriptions in Italy (*14*) to a 6% increase in the United States (*15*), both over the 4-month period following vaccination. Published estimates of the impact over entire populations (all ages, vaccinated and unvaccinated, i.e., incorporating both direct and indirect protection) range from 11.3 fewer prescriptions per 1000 person-years in Ontario, Canada (*16*) to 3.9 fewer in South Africa and Senegal (*17*).

References

1. Fleming DM, Taylor RJ, Haguinet F, Schuck-Paim C, Logie J, Webb DJ, et al. Influenza-attributable burden in United Kingdom primary care. Epidemiol Infect. 2016;144:537–47. PubMed https://doi.org/10.1017/S0950268815001119

2. Hayward AC, Fragaszy EB, Bermingham A, Wang L, Copas A, Edmunds WJ, et al.; Flu Watch Group. Comparative community burden and severity of seasonal and pandemic influenza: results of the Flu Watch cohort study. Lancet Respir Med. 2014;2:445–54. PubMed https://doi.org/10.1016/S2213-2600(14)70034-7

3. Cromer D, van Hoek AJ, Jit M, Edmunds WJ, Fleming D, Miller E. The burden of influenza in England by age and clinical risk group: a statistical analysis to inform vaccine policy. J Infect. 2014;68:363–71. PubMed https://doi.org/10.1016/j.jinf.2013.11.013

4. Pouwels KB, Dolk FCK, Smith DRM, Robotham JV, Smieszek T. Actual versus 'ideal' antibiotic prescribing for common conditions in English primary care. J Antimicrob Chemother. 2018;73(suppl_2):19–26. PubMed https://doi.org/10.1093/jac/dkx502

5. Meier CR, Napalkov PN, Wegmüller Y, Jefferson T, Jick H. Population-based study on incidence, risk factors, clinical complications and drug utilisation associated with influenza in the United Kingdom. Eur J Clin Microbiol Infect Dis. 2000;19:834–42. PubMed https://doi.org/10.1007/s100960000376

6. Pitman RJ, Nagy LD, Sculpher MJ. Cost-effectiveness of childhood influenza vaccination in England and Wales: results from a dynamic transmission model. Vaccine. 2013;31:927–42. PubMed https://doi.org/10.1016/j.vaccine.2012.12.010

7. Baguelin M, Camacho A, Flasche S, Edmunds WJ. Extending the elderly- and risk-group programme of vaccination against seasonal influenza in England and Wales: a cost-effectiveness study. BMC Med. 2015;13:236. PubMed https://doi.org/10.1186/s12916-015-0452-y

8. Pouwels KB, Hopkins S, Llewelyn MJ, Walker AS, McNulty CA, Robotham JV. Duration of antibiotic treatment for common infections in English primary care: cross sectional analysis and comparison with guidelines. BMJ. 2019;364:l440. PubMed https://doi.org/10.1136/bmj.l440

9. European Centre for Disease Prevention and Control. Antimicrobial consumption rates by country. 2019 [cited 2019 Apr 18]. http://ecdc.europa.eu/en/healthtopics/antimicrobial_resistance/esac-net-database/Pages/Antimicrobial-consumption-rates-by-country.aspx

10. Cassini A, Högberg LD, Plachouras D, Quattrocchi A, Hoxha A, Simonsen GS, et al.; Burden of AMR Collaborative Group. Attributable deaths and disability-adjusted life-years caused by infections with antibiotic-resistant bacteria in the EU and the European Economic Area in 2015: a population-level modelling analysis. Lancet Infect Dis. 2019;19:56–66. PubMed https://doi.org/10.1016/S1473-3099(18)30605-4

11. Dolk FCK, Pouwels KB, Smith DRM, Robotham JV, Smieszek T. Antibiotics in primary care in England: which antibiotics are prescribed and for which conditions? J Antimicrob Chemother. 2018;73(Suppl_2):ii2–10. PubMed https://doi.org/10.1093/jac/dkx504

12. Lorenzoni L, Koechlin F. International Comparisons of Health Prices and Volumes: New Findings. 2017 [cited 2019 Jul 10]. https://www.oecd.org/health/health-systems/International-Comparisons-of-Health-Prices-and-Volumes-New-Findings.pdf

13. Buckley BS, Henschke N, Bergman H, Skidmore B, Klemm EJ, Villanueva G, et al. Impact of vaccination on antibiotic usage: a systematic review and meta-analysis. Clin Microbiol Infect. 2019;25:1213–25. PubMed https://doi.org/10.1016/j.cmi.2019.06.030

14. Esposito S, Marchisio P, Cavagna R, Gironi S, Bosis S, Lambertini L, et al. Effectiveness of influenza vaccination of children with recurrent respiratory tract infections in reducing respiratory-related morbidity within the households. Vaccine. 2003;21:3162–8. PubMed https://doi.org/10.1016/S0264-410X(03)00253-6

15. Hoberman A, Greenberg DP, Paradise JL, Rockette HE, Lave JR, Kearney DH, et al. Effectiveness of inactivated influenza vaccine in preventing acute otitis media in young children: a randomized controlled trial. JAMA. 2003;290:1608–16. PubMed https://doi.org/10.1001/jama.290.12.1608

16. Kwong JC, Maaten S, Upshur REG, Patrick DM, Marra F. The effect of universal influenza immunization on antibiotic prescriptions: an ecological study. Clin Infect Dis. 2009;49:750–6. PubMed https://doi.org/10.1086/605087

17. Knight GM, Clarkson M, de Silva TI. Potential impact of influenza vaccine roll-out on antibiotic use in Africa. J Antimicrob Chemother. 2018;73:2197–200. PubMed https://doi.org/10.1093/jac/dky172

Appendix Table. Summary of base-case and alternative estimates for uncertainty analysis for the influenza-attributed GP consultation rate (per 1,000 person-years in England and Wales), prescriptions per influenza-attributable GP consultation, and reduction in influenza cases owing to rollout of LAIV

Age group	Influenza-attributed consultation rate			Prescriptions per consultation		Overall LAIV effectiveness		
	Low	Base	High	Low	Base	Low	Base	High
0–6 m	32.2 (17.4–48.4)	29.7 (23.7–35.9)	73.6 (70.6–76.7)	0.238 (0.203–0.273)	0.597 (0.474–0.719)	0.390 (0.330–0.447)	0.574 (0.501–0.651)	0.694 (0.616–0.767)
6m-4 y	32.2 (17.4–48.4)	29.7 (23.7–35.9)	60.9 (59.2–62.6)	0.238 (0.203–0.273)	0.597 (0.474–0.719)	0.469 (0.433–0.517)	0.663 (0.618–0.714)	0.779 (0.739–0.821)
5–14 y	21.0 (9.87–33.3)	22.1 (17.6–26.7)	38.7 (37.7–39.8)	0.238 (0.203–0.273)	0.588 (0.466–0.708)	0.552 (0.507–0.591)	0.754 (0.709–0.794)	0.855 (0.828–0.885)
15–44 y	10.6 (4.99–16.9)	12.8 (10.2–15.4)	18.8 (18.4–19.1)	0.298 (0.290–0.305)	0.676 (0.536–0.814)	0.280 (0.247–0.321)	0.446 (0.394–0.502)	0.585 (0.526–0.655)
45–64 y	6.68 (3.16–10.6)	12.4 (9.84–14.9)	18.3 (18.0–18.6)	0.357 (0.336–0.377)	0.805 (0.639–0.970)	0.262 (0.227–0.298)	0.423 (0.374–0.484)	0.562 (0.497–0.632)
≥65 y	8.45 (4.06–13.2)	12.2 (9.67–14.7)	5.82 (5.56–6.08)	0.357 (0.336–0.377)	0.857 (0.680–1.03)	0.306 (0.250–0.368)	0.477 (0.397–0.561)	0.608 (0.516–0.692)
Overall	11.8 (6.68–17.3)	14.7 (11.7–17.7)	21.4 (20.9–21.9)	0.313 (0.313–0.313)	0.726 (0.576–0.875)	0.323 (0.289–0.358)	0.494 (0.446–0.549)	0.626 (0.572–0.686)

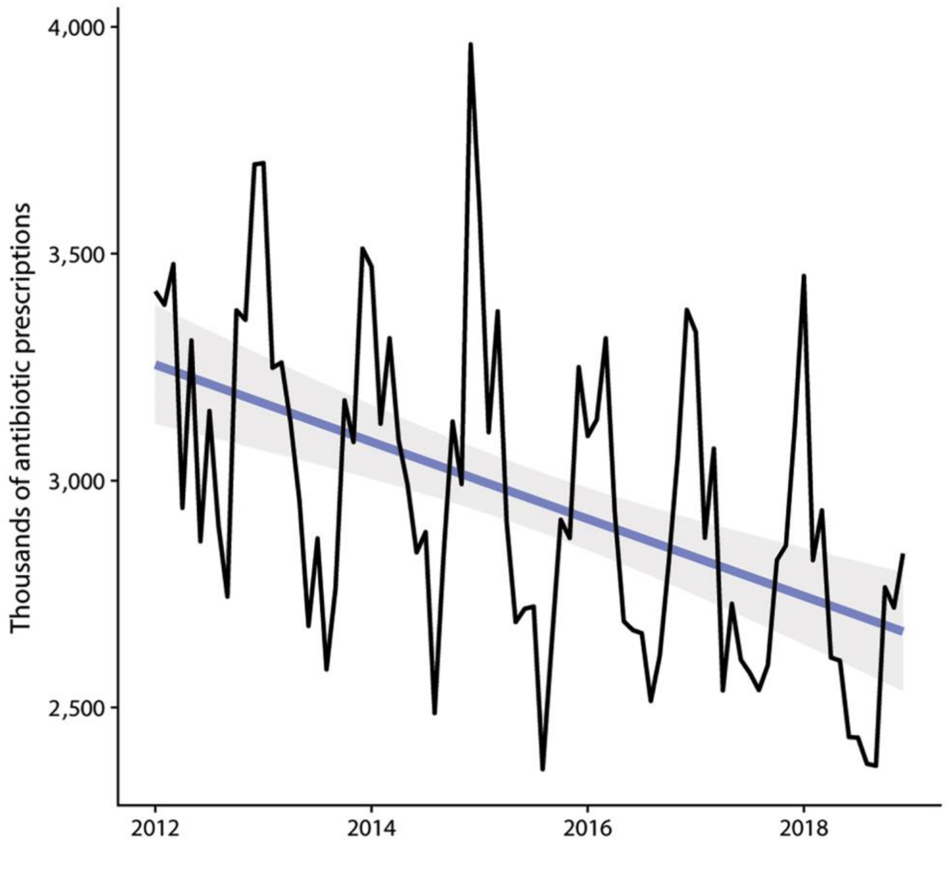

Appendix Figure. Antibiotic use in England has fallen by ≈2.5% each year from 2012 to 2018.
